# Reversibles zerebrales Vasokonstriktionssyndrom: Eine seltene Schlaganfallursache

**DOI:** 10.1007/s00115-024-01674-w

**Published:** 2024-06-06

**Authors:** Enrico Binaghi, Nadine Eube, Susanne Wegener, Anton Schmick

**Affiliations:** 1 Klinik für Neurologie, Universitätsspital Zürich, Rämistrasse 100, 8091 Zürich, Schweiz; 2 Klinik für Innere Medizin, Spital Lachen, Lachen, Schweiz

**Keywords:** Zerebrovaskuläre Störungen, Hirngefäße, Zerebrale Vasospasmen, Donnerschlagkopfschmerz, Auslösende Faktoren, Schlaganfall, Epileptische Anfälle, Neurovascular disease, Cerebral arteries, Cerebrosvascular spasms, Thunderclap headache, Vasospasm triggers, Stroke, Seizures

## Abstract

Das reversible zerebrale Vasokonstriktionssyndrom (RCVS) ist eine komplexe und ätiologisch vielfältige neurovaskuläre Erkrankung, die typischerweise mit Donnerschlagkopfschmerz („thunderclap headache“, TCH) als Hauptkriterium sowie einer reversiblen sekundären Vasokonstriktion der Hirnarterien einhergeht. Das RCVS kann mit oder ohne fokal-neurologische Defizite oder epileptische Anfälle verlaufen. Man unterscheidet zwischen einem idiopathischen RCVS und einem sekundären RCVS, welches durch verschiedene Substanzen, medizinische Eingriffe oder Erkrankungen ausgelöst wird. Den ersten Kontakt mit dieser Erkrankung haben in der täglichen Praxis verschiedene Spezialisten; die richtige Erkennung und Diagnose von RCVS bleiben weiterhin eine Herausforderung. Der klinische Verlauf ist in der Regel monophasisch und selbstlimitierend, wobei Rezidive lediglich in 1,7 % der Fälle pro Jahr auftreten. Komplikationen wie Hirnblutungen und zerebrale Ischämien führen in 5–10 % der Fälle zum Tod. In dieser Arbeit wird ein Fallbeispiel verwendet, um das RCVS und seine Komplikationen vorzustellen sowie die diagnostischen Verfahren zu erläutern.

## Abstract (en)

Reversible cerebral vasoconstriction syndrome (RCVS) is a complex and etiologically diverse neurovascular disorder that typically presents with severe thunderclap headaches (TCH) as the primary symptom, accompanied by reversible vasoconstriction of the cerebral arteries. The clinical course may include focal neurological deficits or epileptic seizures. There are two types: idiopathic RCVS and secondary RCVS, the latter triggered by various substances, medical interventions, or diseases. In clinical practice, various medical specialists may initially encounter this condition, underscoring the importance of accurate recognition and diagnosis of RCVS. The clinical course often appears monophasic and self-limiting, with recurrences reported in only 1.7% of cases annually. Complications such as cerebral hemorrhages and cerebral ischemia can lead to death in 5–10% of cases. This article utilizes a case study to explore RCVS, its complications, and the diagnostic procedures involved.

## Lernziele

Nach Absolvieren dieser Fortbildungseinheit …

- kennen Sie die Diagnosekriterien und Merkmale des RCVS.
- können Sie wichtigsten Auslösefaktoren des RCVS benennen.
- sind Ihnen die Differenzialdiagnosen eines Donnerschlagkopfschmerzes geläufig.
- sind Sie in der Lage, die relevante Diagnostik anzuwenden, um das RCVS zu diagnostizieren.

## Einleitung

Das reversible zerebrale Vasokonstriktionssyndrom (RCVS) ist eine komplexe und ätiologisch vielfältige neurovaskuläre Erkrankung, welche mit der Verfügbarkeit der Magnetresonanztomographie (MRT) häufiger entdeckt wird [1]. Es ist gekennzeichnet durch einen monophasischen Verlauf sowie einmalige oder wiederholte **Donnerschlagkopfschmerzen**

Donnerschlagkopfschmerzen

(„thunderclap headache“, TCH) im Rahmen von zerebralen Vasospasmen. Mögliche Komplikationen sind ein ischämischer oder hämorrhagischer Schlaganfall oder eine subarachnoidale Blutung (SAB), meistens im Bereich der Konvexität des Gehirns.

Bekannte Auslöser sind eine **Schwangerschaft**

Schwangerschaft

(auch ohne Prä‑/Eklampsie), neurochirurgische Eingriffe, der Einsatz vasoaktiver Substanzen, **Triptane**

Triptane,

selektive Serotoninwiederaufnahmehemmer („selective serotonin reuptake inhibitors“,SSRI) Serotonin-Noradrenalin-Wiederaufnahme-Hemmer („serotonin-norepinephrine reuptake inhibitors“, SNRI), der Gebrauch von Drogen (insbesondere Kokain, MDMA [3,4-Methylendioxy-N-methylamphetamin], Speed [Amphetamin]), verschiedene **Immunsuppressiva**

Immunsuppressiva,

**physische Überanstrengung**

Physische Überanstrengung,

**Valsalva-Manöver**

Valsalva-Manöver,

**Orgasmus**

Orgasmus

und **emotionale Ausnahmezustände**

Emotionale Ausnahmezustände

(vgl. Tab. 1).

**Tab. 1:** Auslöser des reversiblen zerebralen Vasokonstriktionssyndroms [2, 25]

*Vasoaktive Medikamente und Substanzen*
Drogen: Cannabis, Kokain, MDMA (3,4-Methylendioxy-N-methylamphetamin), Amphetamine, Lysergsäurediethylamid (LSD)
Antidepressiva: selektive Serotoninwiederaufnahmehemmer (SSRI), Serotonin-Noradrenalin-Wiederaufnahme-Hemmer (SNRI)
α‑Sympathomimetika: z. B. Nasendekongestiva (Phenylpropanolamin, Pseudoephedrin, Ephedrin) und Noradrenalin
CGRP(„calcitonin gene-related peptide“)-Inhibitoren
Transfusionen
Triptane und Ergotalkaloidderivate: z. B. Methergin, Bromocriptin, Lisurid
Orale Kontrazeptiva
COVID-19 („coronavirus disease 2019“)
Nikotinpflaster
Pflanzliche Arzneimittel: z. B. Johanniskraut (*Hypericum perforatum*), Ginseng, Lakritz
Alkoholabusus
Neuroendokrine Tumoren und Phäochromozytom
Immunusuppressiva (Tacrolimus, Cyclophosphamid, Fingolimod, Prednisolon), Erythropoetin, intravenöse Immunglobuline
*Varia*: Kopfverletzung („traumatic brain injury“), subdurales Hämatom, spinales Hämatom, Karotisendarteriektomie, zerebrale Venenthrombose, Liquorhypotonie, autonome Dysreflexie, Hyperkalzämie, Porphyrie, emotionale Situationen, Baden, Husten, Valsalva-Manöver, sexuelle Aktivität
*Postpartum*: mit oder ohne vasoaktive Substanzen, mit oder ohne Eklampsie oder Präeklampsie

### Merke

Das RCVS ist eine komplexe und ätiologisch vielfältige neurovaskuläre Erkrankung, gekennzeichnet durch einen monophasischen Verlauf, abrupte Donnerschlagkopfschmerzen und zerebrale Vasospasmen, häufig ausgelöst durch bestimmte Substanzen oder hormonelle Veränderungen.

## Epidemiologie

Das RCVS betrifft häufiger Frauen als Männer (je nach Studie liegt die Prävalenz zwischen 2:1 und 10:1; [3]). Menschen im Alter von 10 bis 76 Jahren können betroffen sein, die Häufigkeit erreicht ihren Höhepunkt im Alter zwischen 40 und 50 Jahren [3]. In einer amerikanischen Studie betrug die geschätzte jährliche alters- und geschlechtsstandardisierte Inzidenz von RCVS-Hospitalisierungen 2,7 Fälle pro 1 Million Erwachsene [3].

### Merke

Frauen im Alter zwischen 40 und 50 sind am häufigsten betroffen.

### Fallbeispiel

*Die 56-jährige Patientin stellte sich mit seit 2 Tagen bestehendem Benommenheitsgefühl, Schwankschwindel, neuropsychologischer Verlangsamung und starken episodisch-rezidivierenden Kopfschmerzen auf der Notfallstation in einem regionalen Krankenhaus vor. Bis vor 2 Tagen war sie bereits wegen einer Cholangitis und eines exazerbierten chronischen Lumbovertebralsyndroms mit Opiatabhängigkeit im selben Krankenhaus hospitalisiert. Damals erfolgte zur besseren Einstellung der vorbekannten chronischen Schmerzen eine Opiatrotation von Oxycodon und Morphin auf Buprenorphinpflaster. Darüber hinaus wurde eine schmerzdistanzierende Therapie mit Duloxetin mit zusätzlicher Wirkung auf eine vorbekannte depressive Störung initiiert und die Patientin nach Hause entlassen. Auf der Notfallstation war bei der kardiopulmonal kompensierten Patientin der Neurostatus inkl. HINTS(Akronym aus: „head impulse test“/„evaluation of nystagmus“/„test of skew“)-Protokoll mit Lagerungsmanöver unauffällig. Der arterielle Blutdruck zeigte sich erhöht mit systolischen Werten über 170* *mmHg; eine arterielle Hypertonie war bisher nicht bekannt. Die Patientin berichtete über eine selbstständige, gleichzeitige Einnahme von Oxycodon, Morphin und Buprenorphin. Die Symptome wurden initial im Rahmen einer Opiatintoxikation eingeordnet, und die Patientin wurde zur Blutdruck- und Schmerztherapie stationär eingewiesen.*

*Am folgenden Tag war der Schwindel bereits rückläufig. Trotzdem standen seit 2 Tagen zusätzlich starke episodisch-rezidivierende Kopfschmerzen im Vordergrund, ca. 3–4* *h andauernd und ohne komplette Schmerzremission auf Analgetika (Paracetamol und Metamizol neben den oben genannten Opiaten). Die Semiologie der Kopfschmerzen war im Vergleich zur bekannten Migräne mit Aura anders. Ebenso berichtete die Patientin über einen mehrtägigen Konsum von Rizatriptan. Aufgrund der akuten Symptomatik wurde eine native Computertomographie (CT) des Schädels zum Ausschluss einer Hirnblutung veranlasst; diese zeigte keine Auffälligkeiten. Im Verlauf konnten die Kopfschmerzen mittels bedarfsweiser Rizatriptangabe suffizient kontrolliert werden.*

**Abb. 1:**
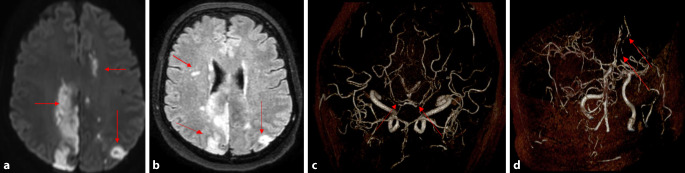
Magnetresonanztomographie (*MRT*) des Neurokraniums; **a,** **b** *DWI* („diffusion weighted imaging“) und FLAIR („fluid-attenuated inversion recovery“) zeigen mehrere multiple frische, allerdings bereits demarkierte Läsionen in mehreren Stromgebieten (*Pfeile*); **c** MRT(Magnetresonanztomographie)-Angiographie mit bilateralen Stenosen der A. cerebri posterior bds. (*Pfeile*); **d** MRT-Angiographie mit bilateralen Stenosen der A. cerebri anterior bds. (*Pfeile*)

*Im Verlauf des stationären Aufenthalts entwickelte die Patientin fluktuierende neurologische Defizite, darunter eine fluktuierende Wahrnehmungsstörung der linken Körperseite, einen Tonusverlust der Extremitäten linksbetont, einen intermittierenden Schwank‑/Drehschwindel und eine neuropsychologische Verlangsamung. Angesichts des fluktuierenden klinischen Bildes ging man initial von einer dissoziativen Störung bzw. von einer neuartigen Aura der bekannten Migräne aus. Um strukturelle Ursachen auszuschließen, wurde eine Magnetresonanztomographie (MRT) des Schädels (kraniale MRT [cMRT]) veranlasst. Hier imponierten neu frische, jedoch bereits in „T1-weighted scan“ und FLAIR („fluid-attenuated inversion recovery“) demarkierte zerebrale Ischämien in multiplen Stromgebieten. Ebenso fielen 2 kurzstreckige Stenosen in der Arteria cerebri anterior im 2. Segment (ACA/2-Segment) links sowie eine kurzstreckige Stenose in der Arteria cerebri posterior im 2. Segment (PCA/2-Segment) beidseits auf *(Abb. 1)*. Bei bereits demarkierten Insulten wurde auf eine Thrombolyse verzichtet. Eine Sekundärprophylaxe mit Acetylsalicylsäure (ASS) wurde begonnen und die Patientin auf eine Stroke Unit einer Universitätsklinik zur weiteren Überwachung und Diagnostik verlegt.*

Fortsetzung des Fallbeispiels im Haupttext (*kursive Passagen*)

## Symptomatik: Schlaganfälle und weitere Komplikationen des RCVS

*Auf der Stroke Unit des Zentrumskrankenhauses präsentierte sich die Patientin mit einem Neglect nach links und einer beinbetonten Hemiparese links, einem NIHSS(National Institutes of Health Stroke Scale)-Score von 5 Punkten entsprechend.*

Bei RCVS sind Schlaganfälle und Hirnblutungen bei etwa 6 % der Patienten im späteren Verlauf der Erkrankung nachweisbar [2, 4, 5]. Sie können einige Tage nach der initial normalen Bildgebung auftreten, wobei die zerebrale Vasokonstriktion angiographisch 2 bis 3 Wochen nach dem klinischen Beginn am stärksten ausgeprägt ist [6].

Zu Beginn zeigen sich, wie im vorliegenden Fall, bei rund 10 % der Patienten **transiente fokal-neurologische Defizite**

Transiente fokal-neurologische Defizite.

Sie dauern zwischen 1 min und 4 h und sind am häufigsten visuell, seltener können sensorische, dysphasische oder **motorische Defizite**

Motorische Defizite

auftreten [7, 8]. Die meisten fokal-neurologischen Defizite haben einen plötzlichen Beginn und sind typisch für diejenigen, die bei transitorischen ischämischen Attacken (TIA) beobachtet werden. Die körperliche Untersuchung ist in den meisten Fällen normal oder zeigt verschiedene fluktuierende Defizite, *womit eine frühzeitige klinische Diagnose eine Herausforderung wird*. In etwa 10–17 % der Fälle sind epileptische Anfälle als frühe Komplikationen des RCVS beschrieben [9]. Nicht selten treten wie im beschriebenen Fall Blutdruckkrisen auf (34–46 %)**.**

### Merke

Frühkomplikationen des RCVS sind Blutdruckkrisen, epileptische Anfälle und transiente fokal-neurologische Defizite. Weitere Komplikationen sind Hirnblutungen und ischämische Schlaganfälle.

Bei Patienten mit RCVS können Defizite im Zusammenhang mit Hirnblutungen oder Ischämien einschließlich Hemiplegie, Aphasie, Hemianopsie oder kortikaler Blindheit persistieren. Das RCVS manifestiert sich in den meisten Fällen mit reinen **Kopfschmerzen**

Manifestation: v. a. Kopfschmerzen

(60–92 %; [10]). Der Verlauf der Erkrankung ist monophasisch ohne neue klinische Defizite nach etwa 3 bis 4 Wochen. Der Donnerschlagkopfschmerz sistiert im Durchschnitt nach 7 Tagen, wobei die anderen neu aufgetretenen Kopfschmerzen im Allgemeinen zirka 3 Wochen nach Beginn verschwinden [11].

### Merke

Der Krankheitsverlauf ist in den meisten Fällen (60–92 %) ausschließlich zephalgisch, monophasisch und verbleibt nach ca. 3 bis 4 Wochen ohne neue klinische Komplikationen.

## Diagnostik und diagnostische Kriterien

### Fallsbeispiel Fortsetzung

*Duplexsonographisch fanden sich eine leichtgradige Atheromatose der extrakraniellen hirnversorgenden Gefäße sowie mehrere segmentale intrakranielle Flussbeschleunigungen in der ACA rechts, in der Arteria cerebri media im 1. Segment beidseits (MCA/1) und in der PCA beidseits. Zur Abgrenzung einer zerebralen Vaskulitis erfolgte eine Liquorpunktion, welche eine normale Zellzahl und Proteinkonzentration ohne Nachweis einer intrathekalen Immunglobulinsynthese ergab. Ebenso blieb ein Vaskulitisscreening im Serum ohne pathologischen Befund. Die kardiologische Abklärung mit transthorakaler und transösophagealer Echokardiographie ergab keine Hinweise auf kardiale Emboliequellen.*

**Abb. 2:**
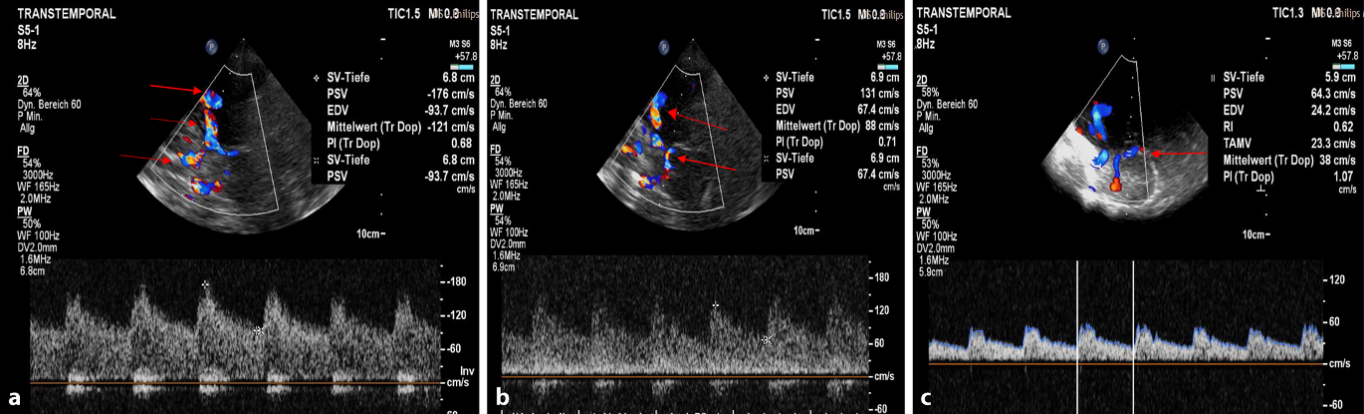
Transkranielle Duplexsonographie: **a,** **b** intrakranielle Flussbeschleunigungen in der A. cerebri media links und rechts sowie in der A. cerebri anterior und posterior links (*Pfeile*); **c** transkranielle duplexsonographische Verlaufskontrolle nach 3 Wochen mit lediglich residualer leichter Flussbeschleunigung in der A. cerebri media links (*Pfeile*) und ansonsten normalen Flüssen in der A. cerebri anterior und posterior links

*Unter Berücksichtigung des fehlenden Wand-Enhancements der basalen Hirnarterien in der Black-blood-cMRT („vessel wall imaging“) mit Kontrastmittelgabe waren die Beschwerden differenzialdiagnostisch mit einem RCVS vereinbar*. *Etwa 3 Wochen später konnte die Verdachtsdiagnose untermauert werden, da die initial bestehende diffuse segmentale intrazerebrale Flussbeschleunigung (in einer transkraniellen Duplexsonographie) bis auf eine entsprechende residuale weniger als 50* *%ige MCA/1-Segment-Stenose beidseits vollständig rückläufig war *(Abb. 2)*.*

Bei den **diagnostischen Kriterien**

Diagnostische Kriterien

des RCVS (Tab. 2) ist die klassische radiologische Präsentation in cMRT oder konventioneller Angiographie eine segmentale Vasokonstriktion mit mindestens 2 Einengungen pro Arterie bei mindestens 2 Arterien („strings and beads“). Die Vasokonstriktion erreicht ihren Höhepunkt am Tag 16 (+/- 10d) und sollte innerhalb von 3 Monaten rückläufig sein, sodass die Diagnose des RCVS letztlich nur retrospektiv bei normalisiertem angiographischen Bild gesichert ist [12]**.** Aufgrund eines wahrscheinlichen initialen Befalls kleinerer distaler Arterien kann die Erstuntersuchung negativ sein. Aus diesem Grund sollte eine **radiologische Verlaufsuntersuchung**

Radiologische Verlaufsuntersuchung

nach 6 bis 27 Tagen (Maximum der Stenosen nach 10 bis 16 Tagen) geplant werden [7, 8, 9, 10, 11, 13, 14]. Weiterhin ist das diagnostische Hauptkriterium des RCVS der Donnerschlagkopfschmerz. Bei unter 60-jährigen Patienten ist ein RCVS die häufigste Ursache für kleine atraumatische **SAB**

Subarachnoidale Blutungen

(SAB) in der Konvexität [13]; somit ist bei den diagnostischen Kriterien zusätzlich ein Ausschluss einer aneurysmatischen SAB entscheidend [2]. Zur Differenzierung von anderen Erkrankungen wie der **primären Angiitis des zentralen Nervensystems (PACNS)**

Primäre Angiitis des zentralen Nervensystems (PACNS)

ist eine Lumbalpunktion sinnvoll. Beim RCVS findet man im Gegensatz zu einer PACNS unauffällige oder nahezu normale Liquorbefunde (Protein < 100 mg/dl, < 15 Zellen/μl), gelegentlich mit einer Blutbeimischung im Fall einer SAB [2]. Typisch für ein RCVS ist der monophasische Verlauf ohne neue Symptome mehr als 1 Monat nach Beginn der klinischen Symptome [14]**.**

**Tab. 2:** Diagnosekriterien für das reversible zerebrale Vasokonstriktionssyndrom [2]

Akute und schwere Kopfschmerzen (oftmals donnerschlagartig) mit oder ohne fokalen Ausfällen oder epileptischen Anfällen
Monophasischer Verlauf ohne neue Symptome mehr als 1 Monat nach Beginn der klinischen Symptome
Segmentale Vasokonstriktion der zerebralen Arterien, nachgewiesen durch indirekte (z. B. Magnetresonanz- oder Computertomographie) oder direkte Katheterangiographie
Keine Hinweise auf eine aneurysmatische Subarachnoidalblutung
Normale oder nahezu normale Liquorwerte (Protein < 100 mg/dl, < 15 Zellen/μl)
Vollständige oder wesentliche Normalisierung der Arterien, nachgewiesen durch eine Folgeuntersuchung mit indirekter oder direkter Angiographie innerhalb von 12 Wochen nach Beginn der klinischen Symptome

### Merke

Die typische Präsentation ist eine perlschnurartige, segmentale Vasokonstriktion mit mindestens 2 Einengungen pro Arterie bei mindestens 2 Arterien („strings and beads“) in einer cMRT-Angiographie, welche innerhalb von 3 Monaten rückläufig ist. Cave: Initial sind die kleinen distalen Arterien betroffen, was zu einer falsch-negativen Beurteilung führen kann. Am 16. Tag nach Symptombeginn erreicht die Vasokonstriktion ihren Höhepunkt und somit steigt die Wahrscheinlichkeit für einen pathologischen Befund in einer MR-Angiographie [13, 14]. Bei holozephalen Donnerschlagkopfschmerzen ist es entscheidend zuerst eine aneurysmatische Subarachnoidalblutung mittels Bildgebung/Liquorpunktion auszuschließen.

## Ätiologie und Pathophysiologie

*Bei der Patientin war außer Rizatriptan ein zusätzlicher Auslösefaktor vorhanden, nämlich der gleichzeitige Beginn der Einnahme des SNRI Duloxetin*.

Etwa 50 % der Fälle von RCVS sind drogen- und medikamentenassoziierte Formen oder peri-/postpartal auch ohne Eklampsie (Tab. 1) bedingt [2, 8, 9]. Etwa 11–13 % der Fälle treten in der Schwangerschaft peri- oder postpartal auf und sind nicht mit einer Eklampsie assoziiert [15]. In den übrigen Fällen liegt kein identifizierbarer Auslösefaktor vor; diese werden als idiopathisch bezeichnet. Etwa 11–25 % der Patienten haben eine Vorgeschichte von arterieller Hypertonie [2]. Es ist jedoch bis jetzt unklar, ob erhöhte Blutdruckwerte, analog zum **posterioren reversiblen Enzephalopathiesyndrom (PRES)**

Posteriores reversibles Enzephalopathiesyndrom (PRES),

eine Störung der Autoregulation der Arterien im Gehirn verursachen und daher Auslösefaktor eines RCVS sein können.

### Merke

Man unterscheidet zwischen einem idiopathischen und sekundären Formen von RCVS.

## Migräne als Differenzialdiagnose

*Bei der Patientin wurde der SNRI Duloxetin gestoppt und eine Basisprophylaxe für die Migräne mit Amitriptylin bei zusätzlicher Wirkung auf die vorbekannte depressive Verstimmung eingeleitet.*

Etwa 17–40 % der Patienten mit RCVS haben zusätzlich eine Vorgeschichte von Migräne [2, 4, 5, 6, 7, 8, 9].

Nicht selten werden in **Notfallsituationen**

Notfallsituationen

die akuten Kopfschmerzen aufgrund von RCVS mit starken Migräneattacken verwechselt [2], obwohl die Patienten die Donnerschlagkopfschmerzen als komplett unterschiedlich von den üblichen Migräneanfällen wahrnehmen [8], somit bleiben eine sorgfältige Anamneseerhebung und anschließend evtl. die Durchführung einer **CT bzw. MRT des Schädels**

CT bzw. MRT des Schädels

zum Ausschluss einer sekundären Genese der Kopfschmerzen entscheidend. Der Zusammenhang zwischen Migräne und RCVS ist noch nicht abschließend geklärt [4]. Eine Akutbehandlung der Migräne mit Triptanen und Ergotaminen kann durch ihre vasokonstriktive Wirkung das Auftreten eines RCVS begünstigen [12, 4], z. B. dann, wenn diese zur Linderung eines Donnerschlagkopfschmerzes gegeben werden, welcher fälschlicherweise für einen Migräneanfall gehalten wird [12]. Blutungskomplikationen treten häufiger bei Frauen und Patienten mit einer vorbekannten Migräne auf [4].

### Merke

Eine sorgfältige Anamnese und klinische Untersuchung helfen in der Differenzialdiagnose zwischen Migräne und neuen, mit RCVS assoziierten Kopfschmerzen.

## Wichtigste Differenzialdiagnose

Die wichtigste Differenzialdiagnose ist die primäre Angiitis des zentralen Nervensystems (PACNS). Bei PACNS sind Männer häufiger betroffen, und der Kopfschmerz ist eher schleichend und kontinuierlich ohne assoziierte Substanzeinnahme oder monophasischen Verlauf. Typisch sind **neuropsychologische Defizite**

Neuropsychologische Defizite

wie Verwirrtheit und langsame demenzielle Entwicklung. Der Liquor ist in den meisten Fällen pathologisch (ca. 80 %) mit einer lymphomonozytären Pleozytose (meist < 20, selten > 100 Zellen) und erhöhtem Gesamtprotein (meist < 120 mg/dl; [16]). Die MRT zeigt diffuse Läsionen der grauen und weißen Substanz in verschiedenem Alter; Blutungen sind bei PACNS eher eine Rarität. Die Angiographie kann die beiden Erkrankungen zwar nicht unterscheiden, eine angiographische Befundnormalisierung nach **intraarterieller Nimodipingabe**

Intraarterielle Nimodipingabe

ist aber diagnostisch hinweisend auf ein RCVS [17, 18] und hilft bei der Abgrenzung vom PACNS.

## Therapie und Prognose

*Im Zentrumskrankenhaus erfolgte bei der 56-jährigen Patientin eine konservative Behandlung mit Beendigung der Applikation vasoaktiver Substanzen und Blutdruckmanagement.*

*Etwa 2 Monate später befand sich die Patientin am Ende des neurologischen stationären Rehabilitationsaufenthalts. Die Wahrnehmungsstörungen und die Hemiparese waren erfreulicherweise unter intensiver Physiotherapie fast vollständig regredient (NIHSS: 1 Punkt) erneute Donnerschlagkopfschmerzen sind nicht mehr aufgetreten.*

Die Therapie ist symptomatisch mit körperlicher Ruhe, Reizabschirmung mit Benzodiazepingabe und intensivem Blutdruckmanagement [2]. Die Nimodipingabe alle 4 h kann gegen Kopfschmerzen helfen, allerdings verhindert sie keine ischämischen Ereignisse und verbessert nicht die Prognose. Prednisolon wird nicht empfohlen, da es zu schlechterem Outcome führt [10]. Die ASS-Indikation besteht lediglich bei hochgradigen Stenosen und zerebralen Ischämien [15]; es kann zur Schmerzkontrolle ebenfalls eingesetzt werden [18, 19]. Auf eine Schmerzkontrolle mittels CGRP(„calcitonin gene-related peptide“)-Antagonisten sollte verzichtet werden, da Fälle beschrieben sind, bei denen dies zur Exazerbation des RCVS beigetragen hat [20, 21]. Eine Schmerztherapie mit Opiaten und Paracetamol oder Ibuprofen scheint kein Risiko für die Exazerbation des RCVS darzustellen. Die Blutdrucktherapie sollte vorsichtig erfolgen, um hämodynamischen Insulten im Rahmen der Vasokonstriktion vorzubeugen. Dies ist insbesondere beim Einsatz von Kalziumkanalblockern zu beachten.

### Merke

Die Priorität liegt darauf, die Anwendung vasoaktiver Substanzen sofort zu beenden. Bei Patienten mit Migräne, die ein RCVS haben, sollte während der Nachbehandlung auf vasoaktive Migränemedikamente (inkl. CGRP-Antagonisten) verzichtet werden. Wie bei unserer Patientin darf man pragmatisch als Basistherapie bei Migräne trizyklische Antidepressiva (Amitriptylin) anwenden, obwohl keine therapeutischen offiziellen Richtlinien für Patienten mit Migräne und RCVS vorliegen [22].

Bei RCVS ist die **Prognose**

Meist gute Prognose

für die meisten Patienten gut; allerdings kann ein schwerer Verlauf eines RCVS, der zu dauerhafter Behinderung oder zum Tod führt, bei 5–10 % der Patienten auftreten [23]. Ein RCVS, das in der peri- oder postpartalen Periode auftritt, erfordert besondere Vorsicht, da es einen fulminanten Verlauf mit multifokalen Infarkten oder intrakraniellen Blutungen mit einem ausgedehnten vasogenen Ödem nehmen kann [24].

## Fazit für die Praxis

- Eine Frühdiagnose des reversiblen zerebralen Vasokonstriktionssyndroms (RCVS) bleibt weiterhin eine Herausforderung. Bei ätiologisch unklaren Schlaganfällen (insbesondere bei Frauen mittleren Alters) sollte an RCVS gedacht werden.
- Das RCVS ist eine wichtige Differenzialdiagnose von Donnerschlagkopfschmerzen und erfordert sofortige Abklärungen, um die Morbidität und Mortalität der Betroffenen zu reduzieren und unnötige oder gefährliche Behandlungen zu vermeiden.
- Ein initial unauffälliger cMRT-Befund schließt ein RCVS nicht aus. Bei einem entsprechenden Verdacht sollte ja nach klinischem Verlauf die zerebrale Bildgebung in 1–4 Wochen nach Symptombeginn wiederholt werden.
- RCVS ist ein seltenes Krankheitsbild; bei neuen und rezidivierenden Vernichtungskopfschmerzen oder bei jedem ischämischen oder hämorrhagischen Hirnschlag mit unklaren intrazerebralen Stenosen ohne kardiovaskuläre Risikofaktoren sollte daran gedacht werden.
- Obwohl das RCVS spontan auftreten kann, insbesondere bei Frauen mittleren Alters, treten mindestens 50 % der Fälle nach Einnahme von vasoaktiven Medikamenten oder nach der Geburt vor; somit ist eine akkurate Anamnese obligat.
- Die Präsentation kann mit neuen epileptischen Anfällen und im Verlauf mit unklaren transitorischen fokal-neurologischen Defiziten kompliziert werden.
- Die primäre Therapie ist rein symptomatisch und besteht aus vorsichtiger Blutdruckeinstellung (mit Vermeidung von Hypotension) sowie Acetylsalicylsäuregabe bei demarkierten Insulten.
- Die Schmerzkontrolle kann mittels Paracetamol, nichtsteroidalen Antirheumatika (NSAR; z. B. Ibuprofen), Metamizol oder Opiaten erfolgen. Die Gabe von Glukokortikoiden ist nicht sinnvoll.

## CME-Fragebogen

Bei welcher Kohorte ist das reversible zerebrale Vasokonstriktionssyndrom (RCVS) am häufigsten ausgeprägt?

Kinder zwischen 5 und 10 Jahren

Männer und Frauen > 80 Jahre

Männer zwischen 40 und 50 Jahren

Frauen zwischen 40 und 50 Jahren

Frauen zwischen 50 und 70 Jahren

In die Notfallaufnahme kommt eine 45-jährige Frau mit seit 3 Tagen wiederholten abrupten holozephalen stärksten Kopfschmerzen über ca. 3 h, wahrgenommen wie ein „Donnerschlag“. Der Neurostatus bleibt ohne pathologischen Befund. Der Blutdruck liegt bei 170/80 mm Hg. Vor 3 Tagen habe sie anamnestisch bei einer Rave-Party Cannabis konsumiert. Welche Maßnahmen führen Sie zuerst durch?

Computertomographie (CT) des Schädels und bei unauffälligem Befund Prednison 50 mg für 5 Tage bei Verdacht auf Status migraenosus

Gabe von Triptanen und bei Besserung der Kopfschmerzen Entlassung nach Hause

CT des Schädels und bei fehlenden Kontraindikationen Liquoruntersuchung und Blutdruckmanagement

Blutdruckmanagement auf dem Notfall und Entlassung nach Hause nach Normalisierung des Blutdrucks

CT des Schädels und bei fehlenden Hinweisen auf Hirnblutung oder Stenose der hirnversorgenden Gefäße Gabe von Acetylsalicylsäure 100 mg als Prophylaxe und Entlassung der Patientin

Welcher diagnostische Parameter ist für das reversible zerebrale Vasokonstriktionssyndrom (RCVS) *nicht *wegweisend?

Normale Liquoruntersuchung

In der Liquoruntersuchung leicht erhöhte Zellzahl (≤ 10 Zellen/µl) und weitere Parameter im Normbereich

Rezidivierende Donnerschlagkopfschmerzen innerhalb von 1 Woche

Segmentale Vasokonstriktion mit ≥ 2 Einengungen pro Arterie bei ≥ 2 Arterien („strings and beads“) in der Magnetresonanztomographie des Schädels (cMRT)

Neue segmentale zerebrale Vasokonstriktion in dedizierter Verlaufsuntersuchung 12 Wochen nach Beginn der klinischen Symptomatik

Bei unter 60-jährigen Patienten ist das reversible zerebrale Vasokonstriktionssyndrom (RCVS) die häufigste Ursache für welche Blutungen?

Aneurysmatische subarachnoidale Blutungen

Intrazerebrale Stammganglienblutungen

Nichtaneurysmatische Subarachnoidalblutungen an der Konvexität

Blutungen zerebraler arteriovenöse Malformationen (AVM)

Spinale Blutungen

Welche Substanz ist ein häufiger Auslösefaktor des reversiblen zerebralen Vasokonstriktionssyndroms (RCVS)?

Acetylsalicylsäure

Nimodipin

Benzodiazepine

Duloxetin

Paracetamol

Welche Maßnahme ist *nicht *bekannt als Auslösefaktor für das reversible zerebrale Vasokonstriktionssyndrom (RCVS)?

Koitus mit Orgasmus und emotionale Ausnahmezustände

Einnahme von Nasendekongestiva

Neurochirurgische Eingriffe wie Karotisendarteriektomie

Einnahme von Immunsuppressiva nach Organtransplantation

Parenterale Substitution bei Vitaminmangel

Eine 40-jährige Patientin, 3 Wochen postpartal, ist seit 10 Tagen auf der Schlaganfallstation hospitalisiert aufgrund bilateraler zerebraler Ischämien und einer kleinen Subarachnoidalblutung noch unklarer Ätiologie im Bereich der Konvexität. Diese wurde in der Magnetresonanztomographie des Schädels (cMRT) am 2. Tag des stationären Aufenthalts festgestellt. Die zu Beginn der Symptomatik stärksten Vernichtungskopfschmerzen sind fast vollständig rückläufig. Die bei Eintritt durchgeführte Computertomographie (CT) des Schädels mit Angiographie der extra- und intrakraniellen Gefäße sowie die transkranielle Dopplersonographie waren ohne pathologischen Befund. Liquoruntersuchung, Vaskulitis und Thromboemboliescreening sind unauffällig. Eine kardiale Schlaganfallabklärung inkl. transösophagealer Echokardiographie und 7‑Tages-Elektrokardiographie (EKG) ergab keine Hinweise auf kardiale Emboliequellen. Was veranlassen Sie als weitere Maßnahme?

cMRT-Angiographie oder konventionelle Angiographie als Verlaufsuntersuchung und bei Nachweis neuer segmentaler Stenosen probatorische Gabe von Nimodipin zur Suche nach einer angiographischen Befundnormalisierung

Pragmatischer Beginn einer oralen Antikoagulation bei Schlaganfällen in mehreren Stromgebieten

Prednisonstoßgabe trotz negativen immunologischen Screenings bei anhaltendem Verdacht auf eine seronegative autoimmune Genese

Keine Verlaufsbildgebungskontrolle und direkt Durchführung einer Hirnbiopsie

Da die Ätiologie des Schlaganfalls bisher unklar ist und die Beschwerden teilweise rückläufig sind, benötigt man keine weitere Diagnostik und belässt die Sekundärprophylaxe mit Acetylsalicylsäure 100 mg täglich

Welche therapeutische Maßnahme ist beim reversiblen zerebralen Vasokonstriktionssyndrom (RCVS) empfohlen?

Acetylsalicylsäuregabe bei lediglich zephalgischem Verlauf ohne hochgradige intrakranielle Stenose

Hoch dosierter Methylprednisolonstoß über 5 Tage

Orale Antikoagulation bei Schlaganfallkomplikationen des RCVS

Körperliche Ruhe, Reizabschirmung, ggf. Gabe von Benzodiazepinen und intensives Blutdruckmanagement

Bei starken Kopfschmerzen schmerzdistanzierende Therapie mit selektivem Serotoninwiederaufnahmehemmer (SSRI) oder Serotonin-Noradrenalin-Wiederaufnahme-Hemmer (SNRI)

Welche Aussage kennzeichnet die Differenzialdiagnose zwischen einem reversiblen zerebralen Vasokonstriktionssyndrom (RCVS) und einer primären Angiitis des zentralen Nervensystems (PACNS)?

Donnerschlagkopfschmerzen sind typisch für beide Erkrankungen.

Subarachnoidale Blutungen sind häufiger bei Patienten mit PACNS als bei Patienten mit RCVS.

Bei beiden Erkrankungen ist in den meisten Fällen die Liquoruntersuchung ohne pathologischen Befund.

Frauen zwischen 40 und 50 Jahren sind bei beiden Erkrankungen am häufigsten betroffen.

Beim RCVS ist der Verlauf der Symptomatik eher monophasisch, beim PACNS ist der Verlauf eher schleichend, kontinuierlich; Auftreten von neuen Beschwerden ist auch nach 12 Wochen möglich.

Was trifft für die Prognose beim reversiblen zerebralen Vasokonstriktionssyndrom (RCVS) zu?

Die Prognose ist für die meisten Patienten gut, allerdings kann eine schwere Behinderung oder Tod bei 5–10 % der Patienten auftreten.

Die Prognose ist besonders günstig im Postpartum.

Die Prognose ist durch die Gabe von Nimodipin mit Reduktion der Inzidenz von ischämischen Ereignissen besser als ohne.

Die Prognose ist bei Frauen mit einer vorbekannten Migräne aufgrund des reduzierten Risikos von Blutungskomplikationen günstiger.

Die Prognose ist aufgrund des erhöhten Rezidivrisikos von über 30 % nicht so günstig.
